# Enhanced Insulation Performances of Crosslinked Polyethylene Modified by Chemically Grafting Chloroacetic Acid Allyl Ester

**DOI:** 10.3390/polym11040592

**Published:** 2019-04-01

**Authors:** Xin-Dong Zhao, Wei-Feng Sun, Hong Zhao

**Affiliations:** Key Laboratory of Engineering Dielectrics and Its Application, Ministry of Education, Heilongjiang Provincial Key Laboratory of Dielectric Engineering, School of Electrical and Electronic Engineering, Harbin University of Science and Technology, Harbin 150080, China; xindong_hrbust@126.com

**Keywords:** crosslinked polyethylene, chloroacetic acid allyl ester, trap state, space charge, chemical graft

## Abstract

Modified crosslinked polyethylene (XLPE) with appreciably enhanced DC electrical insulation properties has been developed by chemical modification of grafting chloroacetic acid allyl ester (CAAE), exploring the trapping mechanism of charge transport inhibition. The bound state traps deriving from grafted molecule are analyzed by first-principles calculations, in combination with the electrical DC conductivity and dielectric breakdown strength experiments to study the underlying mechanism of improving the electrical insulation properties. In contrast to pure XLPE, the XLPE-*graft*-CAAE represents significantly suppressed space charge accumulation, increased breakdown strength, and reduced conductivity. The substantial deep traps are generated in XLPE-graft-CAAE molecules by polar group of grafted CAAE and accordingly decrease charge mobility and raise charge injection barrier, consequently suppressing space charge accumulation and charge carrier transport. The well agreement of experiments and quantum mechanics calculations suggests a prospective material modification strategy for achieving high-voltage polymer dielectric materials without nanotechnology difficulties as for nanodielectrics.

## 1. Introduction

Polymer dielectric nanocomposite–nanodielectrics represent evident improvement of dielectric properties such as breakdown strength in comparison with pure polymer insulation materials. Advanced property optimization can be obtained by controlling polarity of nanoparticle surface in addition to polishing up particle dispersion in matrix [1,2]. It is shown that filling the nanoparticles barely into polymer matrix can prevent conductive percolation across filler/matrix interfaces leading to decreased interface polarizability and enhanced dielectric breakdown strength [3,4]. While the structure and state of nanofiller/matrix interface dominate the dielectric properties, the physical mechanism about how these properties are realized has not been exactly understood for space charge suppression. The nanoparticles filled in the polymer matrix will introduce numerous deep traps for charge carriers at nanofiller/matrix interface, which can capture injected charges to cause electric shielding to further charge injection and as scattering centers to inhibit charge carrier transport, consequently suppressing space charge accumulation [5,6]. Whereas this suppression mechanism depends greatly on the nanoparticle dispersion, such that no significant modification could be achieved unless nanoparticles with a small size are uniformly dispersed in matrix [7]. The adequately large surface/volume ratio is essentially necessary to enhance breakdown strength, while nanofillers tend to agglomerate into larger size, resulting in considerable space charge accumulation and insulation performance degradation. Moreover, it has been found that nanocomposites will absorb significantly more impurities such as water than the unfilled polymers when exposed to ambient environmental conditions due to the presence of nanoparticle/matrix interface which acts as a preferred aggregation position [8]. Therefore, the modification scheme of nanodielectrics always bears the inevitable limitation of nanotechnology, and a novel strategy of suppressing space charge accumulation needs to be urgently developed. N. Quirke has theoretically studied the trap level distribution in polymer material deriving from physical and chemical defects by means of first-principles calculations, rendering a reasonable prediction that polar groups would produce deep traps in polymer materials [9,10]. Y. Zhou reported experimentally the excellent electrical properties of chemically modified polypropylene by grafting with maleic anhydride and analyzed the related mechanism of space charge suppression and enhanced electrical breakdown strength with lower conductivity [11].

In the high interest of fulfilling the promising polymer modification with appreciably improved properties, it is essentially significant to find and verify a novel molecular modification technology to circumvent inevitable impediment in nanotechnology of nanodielectrics. Based on the traps inducing mechanism of space charge suppression in nanodielectrics, a new tactical scheme of introducing deep traps by chemical modification with grafting polar groups is developed so as to avoid the dielectric property deterioration caused by nanoparticle agglomeration. According the suggested routine, we have prepared crosslinked polyethylene grafted with chloroacetic acid allyl ester (XLPE-*g*-CAAE) and systematically studied the obtained electrical properties of pure XLPE and XLPE-*g*-CAAE. The fundamental mechanism of space charge suppression is represented by analyzing the trap level distribution in XLPE-*g*-CAAE. The space charge presence in high-voltage insulation of polymeric cables is demonstrated to account for electric breakdown improvement. The space charge accumulation is correlated with trapping mechanism to comprehend the achieved insulation performance amelioration. General experiment researches concern with the experimental characterization of space charge and with phenomenological models of space charge formation and discharge, while a direct link between molecular properties, space charge formation and eventual breakdown has still to be established. In present paper, the electron bound states intrinsically existing in the CAAE grafted polyethylene (PE-*g*-CAAE) molecule have been calculated using density functional theory (DFT) as implemented in the code DMol3 to analyze the mechanism of suppressing space accumulation and reducing conductivity in combination with experimental results.

## 2. Experiments and Theoretical Calculations

### 2.1. Materials Preparation

Employing Dicumyl Peroxide (DCP, Nobel Company Ltd., Aksu, China) as initiator, the PE crosslinking and CAAE grafting reactions are simultaneously engendered in the polar compound CAAE and primitive low density polyethylene (LDPE) mixture obtained by melt blending method. The primitive LDPE pellets (LD200GH, Sinopec Company Ltd., Beijing, China), 2 wt% DCP, and 0.3 wt% 1010 antioxidant are blended together with individual 0, 0.5and 1.0 wt% CAAE and dried up before being charged with the Torque Rheometer (RM200C, Hapro Company Ltd., Harbin, China). The melt blending process is implemented at 110 °C with a speed of 40rpm in rotary agitator for 15min. The crosslinking and grafting chemical reactions are introduced when the melting blend being heated to temperature 175 °C with the bearing pressure increased to 15 MPa by plate vulcanizing machine. After 35 min the materials are removed to cool down to room temperature. The obtained materials are compressed into molding films with thickness 100, 200, and 300 μm individually. Finally, the film samples are placed in a vacuum drying chamber at 80 °C to short circuit for 48 h, so as to eliminate the internal stress of the samples and fully remove the unreacted CAAE as well as reaction by-products.

### 2.2. Characterization and Measurements

The grafted CAAE on XLPE is characterized by the Fourier-transform infrared (FT-IR) spectra comparatively measured for XLPE and XLPE-*g*-CAAE with different grafting concentration, in the wavelength range of 500–4000 cm^−1^ with 2 cm^−1^ resolution. The pulsed electro-acoustic (PEA) method are utilized to evaluate space charge accumulation of XLPE and XLPE-*g*-CAAE. The tested samples made in cuboid with side length 50 mm and 0.3 mm thickness. The DC electric field of 3 kV/mm is applied to samples at room temperature for 5 min to obtain reference space charge, after which the applied DC electric field is increased to 40 kV/mm and kept for 130 min to achieve space charge accumulation under applied voltage. The short circuit space charge distribution can been finally present by short connection of high voltage electrode to ground electrode.

Thermal extension test is performed with the sample preparation and measurement procedures being in accordance with GB/T 2951.11-2008. Firstly, the dumbbell-shaped specimens with a thickness of 1mm and a middle scaled distance of 20 mm are prepared. Then, the specimens are fixed and hung vertically and weighted according to the standard of 0.2 N/mm^2^. Finally, the specimens are placed in the environment of 200 °C for 15 min. The gauge length is recorded to calculate load elongation. A set of experiments are carried out to calculate the average.

The trap level distribution in XLPE and XLPE-*g*-CAAE are estimated employing thermally stimulated depolarization current (TSDC) measurements. The aluminum film electrodes of 25mm diameter need to be produced by vacuum evaporation on both sides of samples for TSDC measurement. TSDC measurement includes three steps as follows. The samples are polarized under DC electric field of 30 kV/mm at room temperature for 30 min, and then rapidly cooled down to 0 °C holding for 3 min. Finally the applied voltage is removed with the samples being short circuited for 5 min. The TSDC are recorded from 0 to 90 °C with a heating rate of 3 °C/min.

The standard three-electrode system equipped with high resolution digital electro-meter of model KEITHLEY6517B is utilized to measure conduction current in applied DC electric field range of 5–50 kV/mm by step boosting mode with a step of 5 kV/mm. The samples are hot-molded into 200 μm with two surfaces being vacuum evaporated by aluminum plating electrodes of test (diameter 50 mm)-protection (internal diameter 54 mm; external diameter 74 mm) and high-voltage (diameter 76 mm) respectively. The conduction current at every electric field sampling point is recorded as to calculate current density after charging for 130 min to approach to stationary state. The DC dielectric breakdown strength measurement is according to IEC 60243-2-2001 standard experiment setup. The samples of 100 μm thickness and two surfaces vacuum evaporated with aluminum film electrodes of 35 mm diameter are prepared for DC breakdown strength testing. The two measuring electrodes of column shape are used with diameters of 25 mm and 75 mm respectively. The samples with all the electrodes are immersed into silicone oil in measuring process to prevent surface discharge. 15 points are measured for every kinds of material with voltage increasing speed of 2 kV/s.

### 2.3. Molecular Modeling and First-Principles Methodology

The molecular models for representative PE-*g*-CAAE (and the derivative PE-*g*-Allyl Acetate) are initially constructed according to the equilibrium C–C and C–H bond length of 1.50 Å and 1.10 Å respectively with random distributed torsion, in which the PE molecules of 60 polymerization degree are randomly multi-branched with the branch-to-backbone ratio of 1/10 and branch probability of 10% and grafted by CAAE molecule near the middle position of PE backbone chain based on rotational isomeric state (RIS) model. The constructed initial polymer conformations are geometrically optimized to relaxation by total energy functional minimization of first-principles calculations so that the energy change, atomic force and displacement are lower than 1.0 × 10^−5^ eV/atom, 0.03 eV/Å, and 0.001 Å respectively. The electronic structures are calculated for molecular orbitals and electronic density of sates to investigate band-edge features and grafting-introduced trap states. The first-principles calculations are performed employing the scheme of all-electron and numerical atom-orbitals as implemented in DMol3 program of Materials studio 8.0 software package [12], as the detailed methodology adopted in calculations listed in Table 1. DMol3 is a professional calculation code of modeling the electronic structure and energetics of molecules, solids, and surfaces using density functional theory, which produces highly accurate results while keeping the computational cost fairly low for an ab initio method.

## 3. Results and Discussion

### 3.1. Graft and Crosslink Structure

**Figure 1:**
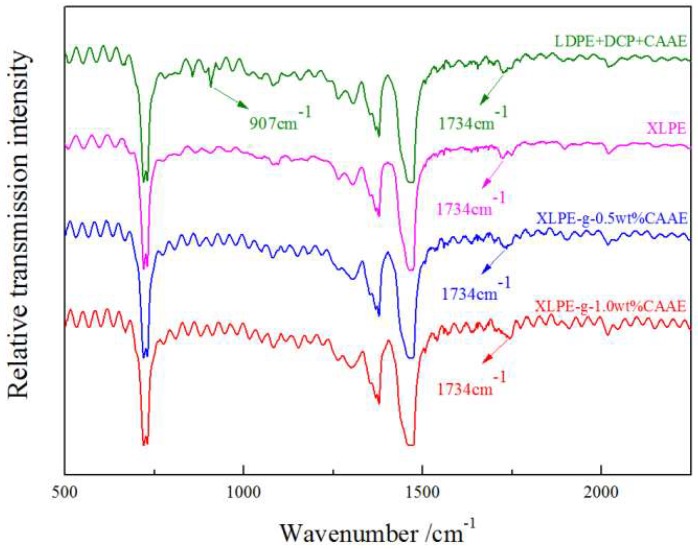
Fourier-transform infrared (FT-IR) of crosslinked polyethylene (XLPE), LDPE + Dicumyl Peroxide (DCP) + chloroacetic acid allyl ester (CAAE) and crosslinked polyethylene grafted with chloroacetic acid allyl ester (XLPE-*g*-CAAE).

The molecular presence on chemically grafted XLPE can be verified with esters C=O vibration from IR spectroscopy, especially for grafted CAAE with characteristic stretching vibration peaks at 1734 cm^−1^, as shown in Figure 1. The newly appearing C=O peak intensity of XLPE-*g*-CAAE increases with the CAAE grafting concentration, to the highest value for 1.0 wt% XLPE-*g*-CAAE. Moreover, the characteristic C=C infrared absorption peak at 907 cm^−1^ of CAAE monomer arises in the mixtures of LDPE, DCP, and CAAE without crosslinking and grafting reactions. However, the peak does not arise in XLPE-*g*-CAAE, demonstrating that CAAE has been grafted on the molecular chain of XLPE. The FTIR results indicate explicitly that the CAAE has been substantially grafted to XLPE for each grafting concentration.

The elongation values indicate inversely the crosslinking probability of XLPE atomic chains, representing the crosslinking reduction resulting from grafting CAAE, as listed in Table 2 presenting thermal elongations of XLPE and XLPE-*g*-CAAE. As graft active centers, the DCP are consumed by grafting CAAE but the crosslinking reactions between the macromolecular chains are not inhibited in the crosslinking process. Grafting CAAE has not decreased the thermal mechanical property and the prepared XLPE-*g*-CAAE are qualified to heat resistance in electrical insulation.

### 3.2. Space Charge Characteristics

**Figure 2:**
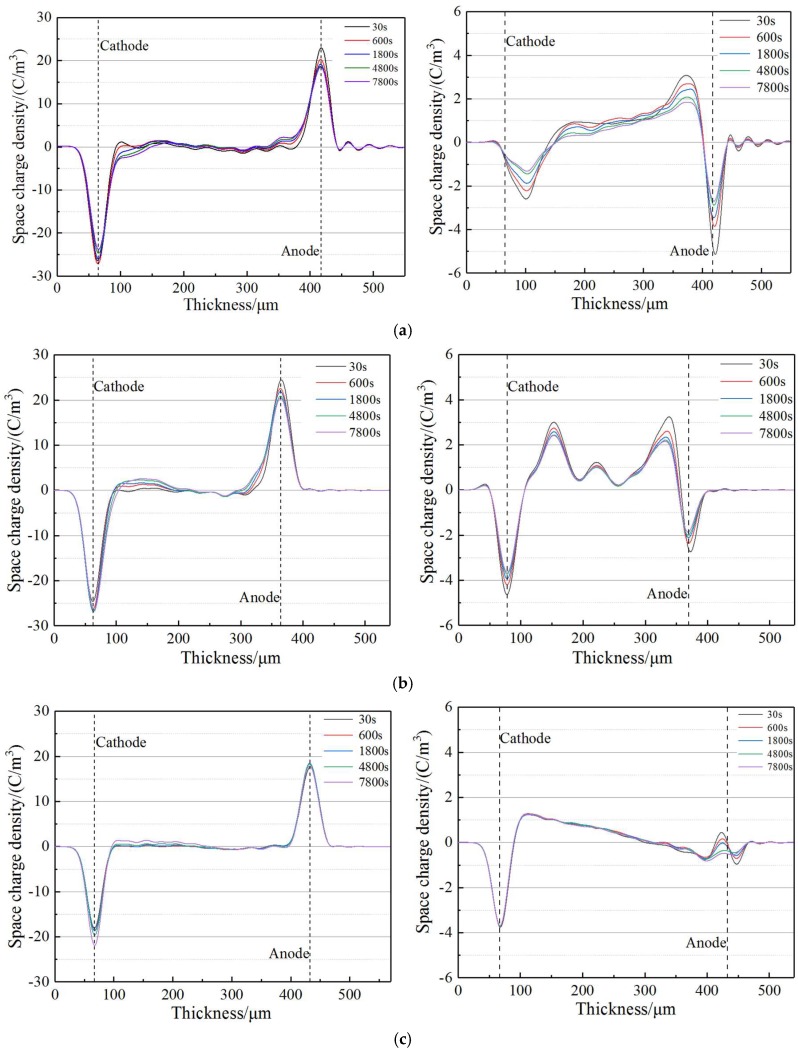
Space charge distribution in (**a**) XLPE, (**b**) XLPE-*g*-0.5wt%CAAE, and (**c**) XLPE-*g*-1.0wt%CAAE under applied DC electric field 40 kV/mm (left panels) and with electrode short connection (right panels).

The space charge distributions of XLPE and XLPE-*g*-CAAE at ambient temperature are measured with PEA method [13], as exhibited in Figure 2 presenting space charge distribution after polarization under applied DC electric field of 40 kV/mm for 130 min (left) and then with electrode short connection (right). It is noted from right panel of Figure 2a that homocharges have been evidently accumulated near both cathode and anode in pure XLPE, with the space charge injection depth increasing with polarization time. In comparison as explicitly shown in left panels of Figure 2b,c for XLPE-*g*-CAAE, the 1.0 wt% CAAE-grafting significantly reduces space charge accumulation so that only small quantity of heterocharge accumulate at cathode with remarkably decreased homocharges accumulation at anode, while the heterocharge accumulation at cathode for 0.5 wt% CAAE-grafting is evidently aggravated so as to exacerbate internal space charge accumulation. Especially for the XLPE-*g*-1.0wt%CAAE, the minimum total quantity of space charges which are mainly distributed near electrodes, has been obtained.

The space charge distributions with electrode short connection as shown in right panels of Figure 2 illustrate that heterocharge injection has been considerably inhibited in XLPE-*g*-CAAE when the CAAE-grafting concentration increases to 1.0 wt%, representing as the accumulated space charge quantity is observably abated to lower than 0.46 C/m^3^ near anode. It is noted for pure XLPE that an appreciable amount of homocharges appear near both cathode and anode with peak values of 2.59 C/m^3^ and 3.08 C/m^3^ respectively, which decrease to 1.31 C/m^3^ and 1.83 C/m^3^ after 7800 s. In comparison, XLPE-*g*-0.5wt% CAAE sample shows higher density of heterocharges and homocharges at cathode and anode respectively with peak values of 3.00 C/m^3^ and 3.22 C/m^3^, which decrease in higher speed to 2.34 C/m^3^ and 2.07 c/m^3^ respectively after 7800 s. The space charge accumulation inside XLPE-*g*-0.5wt%CAAE is not suppressed due to that the density of grafted CAAE is not sufficient to introduce compactly distributed deep traps which can form an effective shielding layer to impede charge injection near electrode. Even, the deep traps presented by grafted-CAAE inside material can capture the injected charge to increase space charge accumulation of internal region. Therefore, the low grafting specimen of 0.5 wt% XLPE-*g*-CAAE however represent deteriorated space charge distribution. Nevertheless, for XLPE-*g*-1.0wt%CAAE sample, only a small amount of heterocharge and homocharge are found near cathode and anode with peak values of 1.26 C/m^3^ and 0.46 C/m^3^ respectively, almost retaining unchanged heterocharge until 7800 s. The space charge measurement results demonstrate that significant space charge suppression can be achieved in XLPE-*g*-CAAE with substantial grafting concentration and suggest a reasonable insulation application for high-voltage DC cable.

### 3.3. Electric Charge Trapping

**Figure 3:**
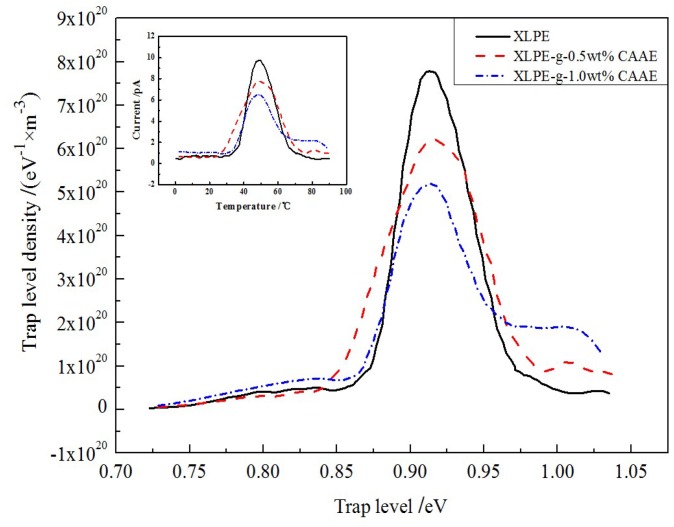
Thermally stimulated depolarization current (TSDC) spectrum (inset panel) and derived trap levels distribution of XLPE and XLPE-*g*-CAAE.

**Figure 4:**
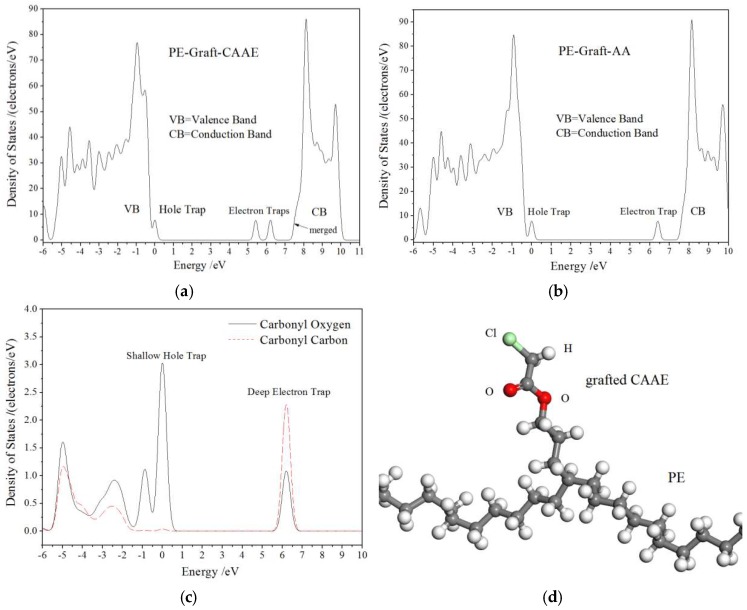
First-principles calculated (**a**) density of state (DOS) of CAAE grafted polyethylene (PE-*g*-CAAE, (**b**) DOS of allyl acetate-grafted-PE (PE-*g*-AA), (**c**) projected density of states (PDOS) on the oxygen and carbon of C=O in PE-*g*-AA, (**d**) schematic molecular structure in relaxation (after geometry optimization) of PE-*g*-CAAE. The highest occupied molecular orbital (HOMO) is referenced as energy zero and smearing is 0.1 eV.

Electric charge carrier trapping mechanism of polymer materials has essential correlation to the space charge accumulation and charge transport [14]. Therefore, trap energy levels need to be evaluated so as to understand the space charge suppression mechanism in XLPE-*g*-CAAE, which can be precisely calculated by modified TSDC theory from the TSDC measurement [15,16]. The TSDC testing temperature range of 0–100 °C is adopted pertaining to thermal excitation of trapped charges for both PE intrinsic traps and grafting-introduced deep traps, in which the practical ambient temperatures of HVDC cable in operation are also included. The TSDC spectra and derived trap level distributions of XLPE and XLPE-*g*-CAAE are presented in Figure 3, indicating grafting-introduced deeper traps of 1.01–1.03 eV in XLPE-*g*-CAAE which are nicely confirmed in accordance with our theoretical results from first-principles calculations as illustrated by density of state (DOS) in Figure 4a. N. Quirke et al. reported theoretically by molecular simulation that the chemical carbonyl functions will introduce deep trap levels of 0.90eV [10], while Y. Zhou reported the higher results of 1.0eV in experiment which are well consistent with our experimental results [11]. The energy depth and density of trap levels in XLPE-*g*-CAAE are listed in Table 3. The 1.0 wt% CAAE grafted XLPE represents the maximum intrinsic trap density and the deeper introduced trap level. The deepest trap level peaks of XLPE, 0.5 wt% and 1.0 wt% XLPE-*g*-CAAE are 0.90, 1.01, and 1.03 eV with trap level densities of 7.75 × 10^20^, 1.01 × 10^20^ and 1.99 × 10^20^ (eV^−1^·m^−3^) respectively, indicating that significantly numerous deeper traps have been introduced by grafting CAAE with the trapping depth slightly rising with increasing concentration of grafted radical.

The considerably reduced space charge accumulation in XLPE-*g*-CAAE can be attributed to the uniformly distributed deep traps introduced by grafting CAAE. The deep traps with evidently uniform density in XLPE-*g*-CAAE will effectively capture and fix the injected heterocharges and forms homogeneous electrostatic charge shielding layer under applied DC electric field. Consequently, the electrostatic shielding area near the electrodes will remarkably inhibit further charge injection, where the trapped heterocharges represent as blocking effect to charge injection and impede charge carrier transport. Therefore, the space charge accumulation in XLPE-*g*-CAAE is significantly suppressed due to the deep traps introduced by grafted polar groups of CAAE. Furthermore, grafting introduced traps 0.11eV deeper than intrinsic traps can retain the highly-efficient suppression mechanism of space accumulation at the temperature approaching to 85 °C which is significantly higher than the 55 °C for pure XLPE without grafting. These results suggest that XLPE-*g*-CAAE can persist in enhanced insulation performance at the experimental temperature high to 85 °C.

TSDC test detect the current produced from thermally excited charges which have been trapped by bound states of electron or holes, in which the peak value at a temperature pertains to the whole quantity of charges trapped throughout the material for a certain trapping depth. The higher density space charges deriving from charge capture by grafting-introduced deep traps only accumulate in a very thin layer region near to electrode. Accordingly, the a new peak in XLPE-*g*-CAAE TSDC spectra arises at a higher temperature signifying larger tapping depth with the peak intensity increasing as grafting density increases, while the lower temperature peak corresponding intrinsic traps from PE defects is reduced, which confirms the space charge suppression by chemically grafting CAAE.

As shown in Figure 4a, our first-principles DOS results predict that the CAAE-grafting can introduce one shallow hole trap state (occupied bound state with intrinsic energy level close to VB band-edge) and two electron trap states (unoccupied bound states with intrinsic energy levels near to CB band-edge in band-gap) in the PE-*g*-CAAE system. The energy levels of these bound states in band-gap described as trap level depths are listed in far right column of Table 3, in which 1.0 eV electron trap level is well comparable with our experimental TSDC results. In order to investigate which polar bonding atoms in the grafted CAAE account for the produced deep trap states, the first-principles calculations on allyl acetate-grafted-PE (PE-*g*-AA) with absent chlorine as deleted from PE-*g*-CAAE are also performed for electronic structure in ground state, as the DOS results shown in Figure 4b. It is indicated from comparison between Figure 4a,b that the second electron trap state completely vanishes for PE-*g*-AA without polar-bonding chlorine atom, while the hole trap and first electron trap states retain unchanged in both energy level and density, demonstrating the deeper 1.8 eV trap presentation by chlorine of grafted CAAE which needs experimental confirmation with more higher temperature TSDC. Further, it can be verified from the projected density of states (PDOS) on the specific atoms of polar-bonding carbonyl (C=O) that the shallow hole trap sate and the first electron trap state origin primarily from oxygen and carbon in polar C=O segment, respectively. The molecular modeling of PE-*g*-CAAE after being relaxed by geometry optimization is present in Figure 4d.

### 3.4. Electric Conductivity and Breakdown Strength

**Figure 5:**
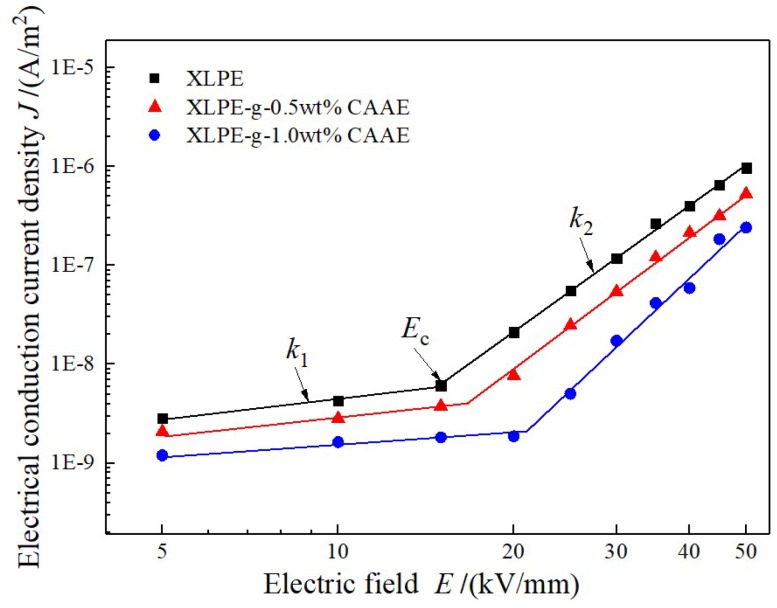
Electrical conduction current density of XLPE-*g*-CAAE varying with applied electric field.

The conduction current density *J* depending on varying applied DC electric field E are measured to investigate the effect of CAAE-grafting-introduced deep traps on the charge carrier transport characteristics in XLPE-*g*-CAAE. The *E*–*J* curves of both XLPE and XLPE-*g*-CAAE represent as two distinct rising stages, as shown in Figure 5. The dependence of conduction current density *J* varying with electric field strength *E* can be represented as:

(1) $$J=AE^{k}$$

and the logarithm with 10 as base (lg) be formularized by:

(2) lg(J)=lg(A)+k⋅lg(E)

where the A denotes the factor attributed to sample properties and size and *k* represents the nonlinear coefficient. The lg(*J*) is proportional to lg(*E*) with the gradient *k* describing the nonlinearity of material electrical conductivity [17,18]. In bi-logarithm coordinates, the pure XLPE and XLPE-*g*-CAAE all exhibit two stages of almost perfect linear relationship of the conduction current density increasing with applied electric field in relatively smaller and larger gradients at lower and higher applied electric field respectively, rendering a critical turning point at a demarcated electric field. The slopes at lower and higher electric field *k*_1_, *k*_2_, and the critical electric field *E*_c_ as described nonlinear parameters are listed in Table 4. The electric resistivity and critical electric field of XLPE-*g*-CAAE increases apparently with CAAE grafting concentration, respectively approaching to one magnitude and 45% higher of 1.0 wt% CAAE grafted XLPE than those of pure XLPE, reasonably suggesting the promising low dielectric loss and high working electric field adequate for most practical applications including HVDC cables.

The *E*–*J* curves characteristics will generally represent as based on space-charge limited current (SCLC) theory [19], according to which the conduction current in solid dielectrics obeys the Ohm’s law (*k*_1_ = 1) at lower applied electric field while will increase intensively complying with the Child’s law [20] (*k*_2_ = 2) when the applied electric field grows exceeding critical point. The Child’s law describes *E*–*J* as that no traps exist or all traps are occupied, actually without considering the trapping/detrapping process for conduction current under varying applied electric field. Therefore, the present *E*–*J* curves of XLPE and XLPE-*g*-CAAE exhibit characteristic trap-limited SCLC performance with *k*_2_ distinctly greater than 2 deriving from deep traps, indicating charge carrier traps existing in all samples and remarkably higher trap density of XLPE-*g*-CAAE than XLPE. Furthermore, our first-principles calculated DOS illustrates that the graft-induced electronic bound states with low density energy levels very near to the PE conduction band bottom are merged into conduction band and represent as new band-edge determining the electron charge carrier transport. The new band-edge states have the intrinsic nature of bound state with the wave function substantially being localized around the correlated atoms in grafted molecules and thus render considerably reduced electronic mobility compared with neat PE under low carrier injection, in consistence with the experimental results of remarkably decreased electrical conductivity from grafting CAAE. The deeper traps in XLPE-*g*-CAAE than XLPE can capture charge carriers to lower energy levels being effective at higher temperature, and decrease conduction current with higher the critical electric field of *E*–*J* curves, agreeing well with the TSDC results. Furthermore, compared with pure XLPE, the conductivity of XLPE-*g*-CAAE, although increasing with electric field at a higher rate, is lower and decreases with grafting density in the testing range of electric field that exceeds the upper limit of HVDC cable working ambience. Therefore, it is definitely proved that the impedance to carrier transport has been achieved by chemically grafting CAAE onto the molecular chain of the XLPE applied for HVDC cables.

**Figure 6:**
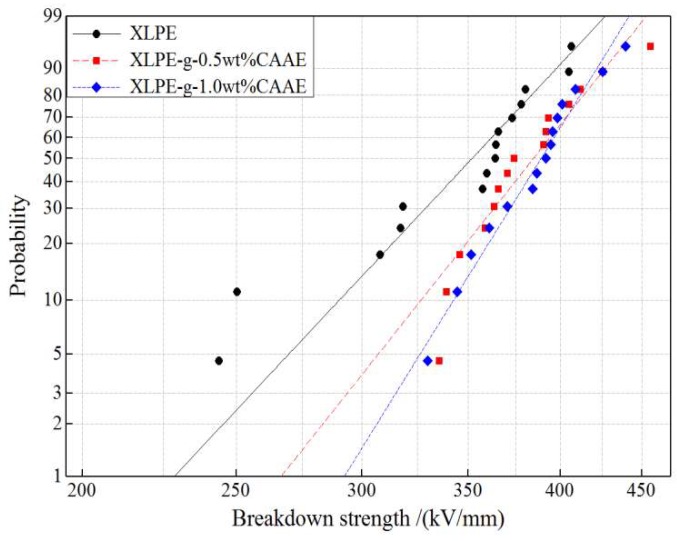
DBS statistics with fitted 2-parameter Weibull distribution for XLPE and XLPE-*g*-CAAE.

The dielectric breakdown strength (DBS) testing results for all the XLPE and XLPE-*g*-CAAE are analyzed by Weibull statistics as provided with 2-parameter Weibull fitting in Figure 6 [21]. The 63.2% probability cumulative DBS together with fitting parameters of Weibull distribution for all the XLPE and XLPE-*g*-CAAE are listed in Table 5. The evident DBS enhancement can be presented by the field strength at 63.2% breakdown probability calculated from scale and location parameters of the fitted Weibull distribution. The chemically grafted XLPE exhibit remarkable DBS improvement directly compared with neat XLPE, demonstrating the evident deep-trap effect deriving from C=O group in grafted CAAE. The XLPE-*g*-CAAE give 9.1% higher of characteristic 63.2% DBS compared with pure XLPE, which is attributed to the deep traps introduced by CAAE graft, capturing charge carriers and suppressing space charge accumulation under DC electric field. Furthermore, it is explicit that the CAAE grafting concentration has substantially affected the DBS. Although the XLPE-*g*-CAAE samples with different concentrations of grafted CAAE have almost the same characteristic DBS, the Weibull shape factor notably increases with CAAE grafting concentration, indicating the DBS can be significantly improved by raising grafted CAAE concentration, as shown in Table 5. The chemical modification effect of CAAE graft on DBS is still retained for the low breakdown probabilities as shown in Figure 6. Comparing the DBS results, it is reasonable to correlate DBS improvement with the increment of trap level depth and density. Those results support the electron trapping model of the grafting polar group and demonstrate that the CAAE is mostly expected as chemically grafting molecule to improve dielectric properties of polymer materials. The polar C=O group in grafted CAAE accounts essentially for DBS improvement of XLPE-*g*-CAAE. Further evidence is shown that the candidate molecules with higher polarity or multiple polar group have been grafted to polymers for chemical modification so as to further increase DBS.

## 4. Conclusions

The significant electric-insulation improvement and the corresponding modification mechanism of chemical grafting with CAAE on the dielectric properties of XLPE have been systemically investigated. The XLPE-*g*-CAAE materials represent excellent insulation performance such as the suppressed space charge accumulation, decreased conduction current density, enhanced critical electric field of *E*–*J* curves, and improved dielectric breakdown strength. The modified dielectric properties of XLPE-*g*-CAAE are attributed to the trapping mechanism of the deep traps produced from the polar groups of grafted CAAE, which can remarkably impede the charge carrier transport and inhibit the space charge injection by electrostatic shielding of deep traps occupied with captured charge carriers. This study demonstrates and suggests a novel strategic scheme to design and realize promising high performance HVDC insulation materials without nanotechnology difficulties of nanodielectrics.

## Figures and Tables

**Table 1:** Methodology and parameters adopted in first-principles calculations as DMol3.

Electronic Hamiltonian	Scheme	Condition and Parameter
Exchange-correlation energy	Meta-generalized-gradient approximation	M11-L
Integration accuracy		2000 grid points/atom
SCF	Tolerance	1 × 10^−6^ eV/atom
	Multipolar expansion	Octupole
	Charge density mixing	Charge = 0.3, DIIS = 5
Core treatment	All Electrons	
Numerical basis set	DNP	Basis file 4.4
Orbital cutoff	Global	3.7 Å

**Table 2:** Thermal extensibility of XLPE-*g*-CAAE.

Materials	Elongation/%
XLPE	22.5
XLPE-*g*-0.5wt%CAAE	22.5
XLPE-*g*-1.0wt%CAAE	22.5

**Table 3:** Trap parameters for XLPE-*g*-CAAE.

Materials	Peak Density/eV^−1^·m^−3^	Trap Level/eV	Calculated Trap Level/eV
XLPE	7.75 × 10^20^	0.91	Electron: 1.0, 1.8; Hole: 0.3
0.5 wt% graft	1.01 × 10^20^	0.92, 1.02	
1.0 wt% graft	1.99 × 10^20^	0.91, 1.03	

**Table 4:** Nonlinear parameters of XLPE-*g*-CAAE.

Materials	*E*_c_/(kV/mm)	*k*_1_	*k*_2_
XLPE	14.9	0.691	4.242
XLPE-*g*-0.5wt%CAAE	16.6	0.624	4.420
XLPE-*g*-1.0wt%CAAE	21.1	0.330	5.462

**Table 5:** The characteristic 63.2% dielectric breakdown strength (DBS) and Weibull distribution fitting parameters in 95% confidence interval. The scale and shape factors are denoted as *E*_b_ and *β* respectively.

Samples	2-Parameter	
	*E*_b/_(kV/mm)	*β*
XLPE	365.4	9.813
XLPE-*g*-0.5wt%CAAE	396.7	11.69
XLPE-*g*-1.0wt%CAAE	398.7	14.86
